# Different-sex American couples’ stress, uncertainty, and fertility desires during the COVID-19 pandemic

**DOI:** 10.1186/s41118-025-00257-0

**Published:** 2025-07-01

**Authors:** Karen Benjamin Guzzo, Alexandra VanBergen, Wendy D. Manning, Claire Kamp Dush

**Affiliations:** 1 The University of North Carolina at Chapel Hill, Chapel Hill, USA; 2 The University of Minnesota, Minneapolis, USA; 3 Bowling Green State University, Bowling Green, USA

**Keywords:** Fertility, Uncertainty, Couples, NCHAT

## Abstract

The Narratives of the Future (NofF) framework has drawn attention to the role of subjective well-being and uncertainty as key determinants of individual fertility intentions. We apply the NofF framework to the Traits–Desires–Intentions–Behavior (TDIB) model, arguing that perceptions of current and future well-being are aspects of traits and thus that desires—not intentions—would be most strongly related to perceptions. Further, although most research on subjective well-being and uncertainty has focused on economic aspects, a life course perspective suggests that other domains, such as health or relationship concerns, are also relevant. Finally, few studies consider the dyadic nature of fertility decision-making. We address these gaps by using the U.S.-based National Couples’ Health and Time Study (NCHAT), collected during the COVID-19 pandemic, to investigate how subjective concerns across economic, health, and relational domains relate to American couples’ agreement on wanting a(another) child and how men’s and women’s own fertility desires are related to their own stress and their partner’s relative stress across different domains. We find that couples’ higher levels of stress—across domains—is related to greater couple-level uncertainty and disagreement about fertility desires. Women’s own fertility desires are associated with their partner’s relative stress across domains, with less evidence that men’s fertility desires are related to their partner’s relative stress. Our findings point to the importance of considering stress and uncertainty across multiple domains, at least during the COVID-19 pandemic, as important for the formation of fertility desires as well as the need to incorporate both partners’ experiences as key factors in fertility decision-making.

## Introduction

As birth rates in the U.S. and elsewhere have fallen in the years following the Great Recession, and more countries have fallen—and stayed—well below replacement-level fertility (Hellstrand, et al., 2021), understanding the drivers of contemporary fertility patterns has taken on new importance. Although the initial declines observed during and immediately after the Great Recession were expected (Sobotka et al., 2011), that fertility rates did not recover in the U.S. as economic indicators showed improvement remains a puzzle (Comolli et al., 2021; Kearney et al., 2022). The COVID-19 pandemic introduced yet another shock to fertility behaviors, with U.S. fertility rates dipping dramatically at the start of the pandemic before a slight recovery to resume the pre-pandemic downward trajectory (Osterman et al., 2024). Certainly, shifts in fertility behavior during the pandemic must be understood in the context of the longer-term trend that emerged after the Great Recession (Comolli, 2023), and the fact that scholars have been unable to fully explain the post-Recession declines presents a challenge.

Prior to the Great Recession, the U.S. had enjoyed comparatively high fertility rates (at or near replacement level) relative to other high-income countries (Wu & Marks, 2023) and, as a result, U.S.-based research on the causes of low fertility were rare. The nascent state of U.S.-based work on fertility delays and declines has hampered our ability to understand trends since the Great Recession and the drivers of contemporary low fertility in the U.S. Part of the inability seems to stem from U.S. scholars’ overreliance on traditional demographic theories that emphasize the role of micro- and macro-economic and policy indicators (Becker, 1960, 1992; Butz & Ward, 1979; Easterlin, 1975). However, recent work in high-income contexts points to the potential importance of subjective evaluations of well-being and perceptions of the future as key components of individual fertility decision-making with the Narratives of the Future (NoF) framework (Guzzo & Hayford, 2025; Vignoli et al., 2020a, 2020b). This work has largely focused on economic concerns, though a life course perspective highlights the potential relevance of concerns in other domains, such as health or relationships (Alderotti & Trappolini, 2022; Barber et al., 2019Manning et al., 2022). 

We apply the insights from the NoF framework to the Traits–Desires–Intentions–Behavior (TDIB) model, which explicitly acknowledges that fertility is the result of a multistep cognitive process. In the TDIB model (Miller, 1994; Miller et al., 2004), desires indicate what people would like to do—what they want—and precede their actual plans (intentions). Desires themselves are informed by traits, or motivations. Most work studying fertility decision-making has focused on fertility intentions; fertility desires are an important but overlooked component—it is where ambivalence about childbearing itself is most strongly expressed and the component that is most proximate to uncertainty and stress about one’s current and future circumstances. The TDIB also highlights the importance of couples for understanding fertility behaviors. Due to data limitations, there are few recent studies of couple-level fertility decision-making using U.S. data despite the fact that fewer than one in seven births in the U.S. happen outside of a coresidential union (Brown, 2022). An important predictor of whether a birth happens is whether couples *agree* about their fertility goals (Shreffler et al., 2019). 

In this paper, we use data from the National Couples’ Health and Time Study (NCHAT). NCHAT is a nationally representative study of cohabiting and married Americans interviewed in 2020 and 2021. Importantly, for a subset of the study population, both members of a couple were interviewed, allowing us to examine couple-level agreement on fertility desires (using multinomial logistic regression). Additionally, the data permit us to examine men’s and women’s own fertility desires and how a partner’s characteristics may be associated with one’s own desires (using ordinal logistic regression). 

We build on prior work by addressing how subjective concerns across economic, health, and relational domains relate to American couples’ agreement on wanting a(another) child as well as consider individual-level fertility desires separately by gender. The couple-level models show how couples’ average stress in different domains is associated with fertility desires, and the individual-level models indicate how a partner’s relative stress is linked to fertility desires net of men’s and women’s own stress. This project moves the field forward in several ways. First, we integrate both new and longstanding perspectives (NoF, TDIB, life course) to study fertility decision-making. Second, we focus on fertility desires rather than intentions, which have been overlooked in prior research, and consider ambivalence in desires themselves. Third, we expand beyond economic factors to consider other domains (health and relationship) that could influence fertility decision-making while still accounting for objective characteristics. 

## Background and theory

### Determinants of fertility desires: the importance of subjective perceptions

Identifying the drivers of macro-level fertility patterns requires understanding the individual decision-making that underlies aggregate rates. The Traits–Desires–Intentions–Behavior (TDIB) model (Miller, 1994; Miller et al., 2004) is a useful heuristic. The TDIB model recognizes that fertility results from a multistep psychological sequence, where individuals have certain conscious and subconscious dispositions (i.e., traits or motivations) that influence whether individuals want to have children (i.e., desires). Desires, in turn, lead to firm plans about having children (i.e., intentions) that take into consideration real-life constraints, such as the lack of a partner. A birth (or lack thereof) is the outcome of this process. As Miller (2011) notes, prior demographic work has largely focused on intentions given their proximity to actual fertility, to the neglect of attention to desires. A focus on desires, however, is warranted, particularly in light of theoretical emphases on uncertainty and subjective evaluations of well-being. Another advantage of considering fertility desires, as Miller et al. (2013) argue, is that this is where ambivalence emerges—that people feel both positively and negatively about wanting a child and thus being uncertain about whether they do, in fact, desire to have a(nother) child. 

In two papers conceptualized prior to the pandemic to explain post-Recession declines in Europe for which more standard economic models were insufficient, Vignoli and colleagues introduced the Narratives of the Future (NofF) framework (Vignoli et al., 2020a, 2020b); this work has similarly been applied in U.S. contexts (Guzzo & Hayford, 2025; Manning et al., 2022). The NoF framework argues that uncertainty should now be a key component for understanding contemporary childbearing decision-making. Childbearing is inherently future-oriented, and so how individuals feel about the future informs their feelings and decisions about having a child. Broader social and economic shifts (such as changes in the labor market, changes in union formation and stability, and—during the pandemic—threats to health) affect whether people see their futures as predictable and stable. It further centers the role of individuals’ subjective evaluations of their past, present, and future scenarios as lenses through which people modify their fertility decision-making accordingly. Because parenthood is an irreversible role, the future seems to weigh especially heavy as models of intensive parenting have become pervasive (Ishizuka, 2019) amid high levels of concern over children’s mental health and worries about children’s future financial and career prospects (Minkin & Horowitz, 2023). People seem to take into consideration different aspects of their current situation and likely future as they decide if they will be able to parent in way that will provide the perceived supports and scaffolding necessary to raise successful children. That is, do they currently have the resources—broadly defined to include economic factors, health and well-being, a strong relationship—to meet the expectations of the parent role? 

Drawing from the TDIB model, we might expect that poorer evaluations of one’s current circumstances might either depress fertility desires or lead to ambivalence. And, indeed, evidence supports this argument, finding that characteristics such as perceived employment instability, worries over future job prospects, and generally negative perceptions of the economy are linked to lower fertility goals and more ambivalence (Brauner-Otto & Geist, 2018; Gatta et al., 2022; Guzzo et al., 2025; Lappegård et al., 2022), though other work suggests economic uncertainty is less relevant than more objective characteristics (van Wijk & Billari, 2024). If the actual process of decision-making about childbearing follows the TDIB model, where might the concepts of NofF framework fit? In our estimation, concepts such as uncertainty, stress, or concerns fall within the “traits” component of the TDIB. Some people are more or less risk averse. Some people are more or less future-oriented. Some people experience more or less stress in response to crises. Because traits/motivations influence desires directly—but intentions only indirectly—we suggest that fertility desires may be a fruitful avenue to explore.

### The life course perspective

The life course perspective, with its emphasis on linked lives, points to the dyadic nature of fertility decision-making; an emphasis on couples also appears in some formulations of the TDIB (Miller et al., 2004). Because most fertility in the U.S. occurs within the context of a couple (Brown, 2022), it is likely that the experiences of both members of a couple inform their fertility decision-making process (Brehm & Schneider, 2019). There is a long history of analyses of fertility behaviors using couple-level data (e.g., Bauer & Kneip, 2014; Beckman et al., 1983; Thomson, 1997; Thomson et al., 1990), though some studies use individuals’ reports about partners as proxies for actual reports from partners in the absence of truly dyadic data (e.g., Testa, 2012; Williams, 1994). Proxy reports, however, are not entirely accurate (Stykes, 2018). Much of this literature focuses on agreement about fertility goals and subsequent fertility (e.g., Berrington, 2004; Shreffler et al., 2019; Testa et al., 2014), with considerably less attention to couples’ joint characteristics (including their subjective perceptions of well-being) and how they are linked to couples’ agreement about future fertility.

Fertility decision-making among different-gender couples is complex, with various interpretations of how couples—and men and women within couples—exert influence on each other. On the one hand, women bear more of the costs and burdens of childbearing and rearing. As such, to the extent that a “sphere of interest” rule would give greater weight to women’s characteristics and attitudes (Corijn et al., 1996), we might expect that men’s fertility decision-making is more influenced by their partners in different-gender couples than vice versa. On the other hand, if we consider power more generally, the continued existence of the male breadwinner model (as a social norm, if not always in practice) and men’s higher earnings relative to their partners leads us to expect that men’s characteristics would more strongly influence women’s fertility decision-making than vice versa. Finally, gender equality in fertility decision-making might also exist, with both men and women equally considering their partners’ experiences and characteristics as they form own their fertility goals (the “egalitarian” rule). Among dyadic studies of fertility goals and behaviors more broadly, most find that there are partner effects but with varying conclusions about the role of gender. Some studies find that the male partner’s effects are stronger than the female partner’s effects (Stein et al., 2014; Thomson et al., 1990) while others finding that partner effects are similar (Matias & Fontaine, 2017). Some work also reports that the significance and magnitude of partner effects varies depending on which characteristics are considered (Hutteman et al., 2013; Matera et al., 2023) or across parity (Testa et al., 2014; Thomson et al., 1990). Although it may be unclear whether men or women are more heavily influenced by their partner’s levels of stress and uncertainty when forming their own fertility desires, it does seem likely that a partner’s stress and uncertainty influences how someone feels about having a child. We expect that an individual’s own uncertainty and stress are related to their desires, but also that their partner’s *relative* stress—whether they are more or less stressed than the respondent themselves—are relevant. Having a partner who is more stressed would likely dampen one’s own desire or lead to ambivalent or uncertain feelings about wanting a(nother) child; having a partner with less stress or the same level of stress, though, seems less likely to influence a person’s own fertility desires net of their own level of stress and uncertainty.

Another central tenet of the life course perspective is multidimensionality—that is, that the life course consists of interrelated domains that affect, and are affected by, each other. From a fertility perspective, people evaluate their current circumstances across a range of factors and evaluate whether having (additional) children will improve their well-being (Huinink & Kohli, 2014); stress is a key component of well-being and strongly linked to family behaviors throughout the life course (Avison, 2010). There is a wide body of literature documenting that economic and employment domains are associated with fertility behaviors, some of which has been discussed above. Still other work takes an opportunity costs framework to analyze the potential career and income penalties that people (mostly women) will face by having a child (Bearak et al., 2021; England et al., 2016) or considers how work–family compatibility and conflict are linked to childbearing decisions and behaviors (Shreffler et al., 2010).

Other domains have received less attention but may be no less relevant. During the COVID-19 pandemic, the health domain was likely especially salient for decision-making in other domains. COVID-19 was, of course, a direct—and during the early period of the pandemic, poorly understood—health threat. Accurate information about how it spread and how to limit exposure was sparse, mitigation strategies (such as masks and vaccines) were not always readily available, and politicization of potential mitigation strategies was common (McCormack et al., 2021). There was also differential exposure, susceptibility, and impact across populations (such as those with pre-existing health conditions, frontline workers, and marginalized groups), which elevated the level of concern and confusion (Treskova-Schwarzbach et al., 2021). Further, there may have been direct concerns linked to childbearing, as it was unclear how a COVID-19 infection could affect fetal development (Edlow et al., 2022). Mitigation strategies that disallowed partners or other guests from accompanying pregnant people to doctors’ appointments and delivery were also common (Arora et al., 2020). Not surprisingly, then, research documents that the pandemic was linked to a host of health and related worries and stressors (like experiences of isolation in pregnancy, childbirth, and the newborn phase due to stay-at-home orders) among new parents, pregnant people, and those who were not yet pregnant but desired a child (Burgess et al., 2022; Naya et al., 2021; Raybould et al., 2023; Wright, 2022). Couples’ joint health stress might lower their desires to have a child or be related to greater ambivalence about having a child.

Another domain that has received considerable, but in some ways superficial, attention is that of romantic relationships. Although there is strong evidence about the role of simply having a partnership in forming and achieving fertility goals (i.e., Barber et al., 2019; Guzzo, 2022; Rackin & Bachrach, 2016), far less is known about how the quality of partnership is linked to fertility decision-making or how couples’ evaluations of their relationship quality influence whether they agree about their fertility desires. Given evidence that couples with shared children have lower risks of dissolution, the general assumption is that there is a selection effect—that couples with better relationships go on to have children together (Lillard & Waite, 1993). Studies do indeed find that relationship quality is an important predictor of fertility goals (Rijken & Thompson, 2011; Manning et al., 2022, 2025). Like the economic and health domains, though, the COVID-19 pandemic had some unique implications for relationships. Due to stay-at-home orders and the emphasis on forming “pods” to limit exposure, some couples seem to have accelerated their relationship progression (Jamison & Kanter, 2023). Yet marriage rates were lower as other couples had to delay marriage due to closures of government offices and wedding locales (Westrick-Payne et al., 2022). And while partnered individuals were more likely to describe benefits of being partnered and spending time together during the pandemic rather than downsides, some partnered individuals noted declining relationship quality (Luetke et al., 2020), especially in circumstances where members of the couple had different reactions to the mitigation strategies related to the pandemic (Rosenfeld & Hausen, 2023).

In this paper, we examine fertility desires as a couple-level construct and at the individual level. Our overarching supposition is that understanding fertility desires requires considering multiple dimensions of uncertainty and well-being in addition to accounting for sociodemographic, economic, and family-related characteristics. First, we consider fertility desires and agreement as a couple-level construct related to couple-level uncertainty and stress:

#### Hypothesis 1:

Higher levels of couple-level stress within the domains of economics, health, and relationships will each, separately, be associated with higher couple-level agreement on not desiring to have a(nother) child and with ambivalence or disagreement about desiring a child.

Second, we consider individual-level models (run separately by gender) testing whether individual fertility goals are associated, in part, by their partner’s relative feelings of uncertainty and stress in addition to one’s own levels of uncertainty and stress across three domains:

#### *Hypothesis 2:*

Having a partner who is more stressed or uncertain about a particular domain will be associated with greater chances of an individual not desiring to have a child themselves and of being uncertain about their fertility desires, net of their own absolute levels of stress or uncertainty.

### A note on the COVID-19 pandemic

The data used in this study were collected during the COVID-19 pandemic. There is a large body of work documenting the declines in fertility during the pandemic in the U.S. (and elsewhere)—declines that were greater than those observed generally since the Great Recession. In the U.S., for instance, the number of daily births fell by an average of 0.96% a year between 2000 and 2019, but in 2020, the average number of daily births was 4.06% lower compared to 2019 (Morse, 2021). Similar patterns were observed in other countries, with fertility declining initially during the pandemic followed by a return to pre-pandemic levels and trajectories (Sobotka et al., 2024). During the period under study, pandemic-related restrictions (stay-at-home orders, school and business openings, vaccine availability, face mask requirements) varied significantly over time and across U.S. states, creating considerable uncertainty and confusion. The pandemic dramatically altered people’s lives in multiple domains, and there is research documenting how the pandemic affected fertility decision-making (e.g., Guetto et al., 2022; Raybould et al., 2023; Wright, 2022), with some work using longitudinal data that demonstrate how people revised their pre-pandemic intentions in response to the COVID-19 pandemic (e.g., Manning et al., 2022; Marteleto et al., 2023). Although we acknowledge the unique impacts of the COVID-19 pandemic on society, we join these authors in arguing that the pandemic merely spotlighted the role that uncertainty and stress played in people’s lives (and that uncertainty seems to be coming from more and more sources) and, thus, believe that our findings generalize beyond the pandemic.

## Data and methods

### Sample

**Fig. 1:**
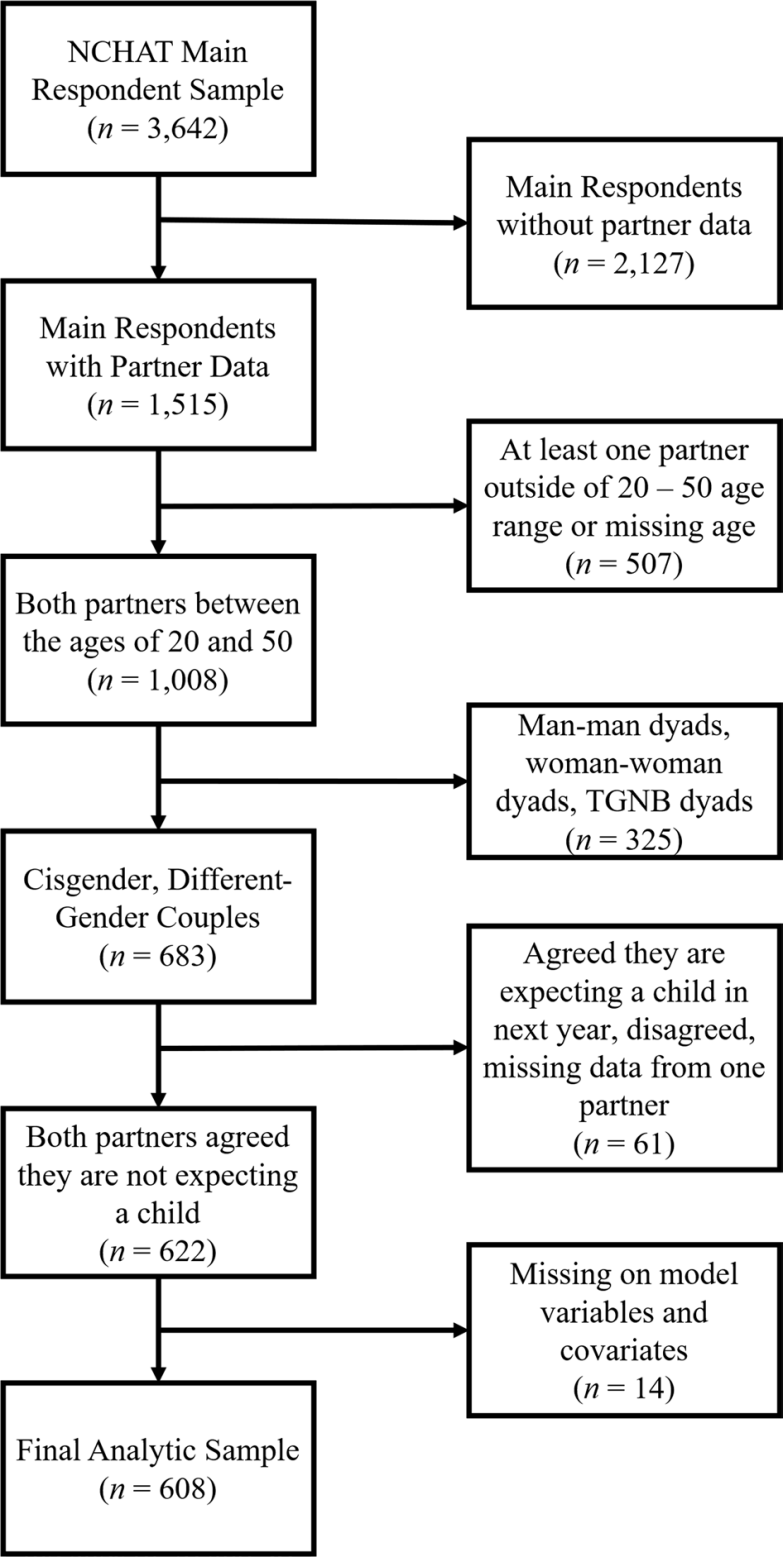
Sample selection

This study uses data from the National Couples’ Health and Time Study (NCHAT), a population-representative sample of cohabiting or married U.S. adults in same- and different-gender partnerships who were between the ages of 20 and 60. Data were collected via an online survey fielded by the Gallup organization between September 2020 and April 2021; for details on the survey design, sampling, and weighting, see Kamp Dush et al. (2023). NCHAT includes survey and time diary data from main respondents and their participating partners. The current study uses survey data from both partners (i.e., dyadic data). Main respondents (*n* = 3,642) were recruited via the Gallup Panel, a population-representative, probability-based research panel with over 100,000 randomly selected U.S. adults who have agreed to participate in research. Main respondents then invited their partners to fill out the same survey (*n* = 1,515). Because our focus is on how partnered men and women’s pandemic-related experiences are linked to their own and their partner’s uncertainty about having a child, we restricted our analytical sample to (a) couples with both partners between the ages of 20 and 50^1^ (excluding 507 dyads); (b) different-cisgender couples (excluding 325 dyads); (c) couples not currently pregnant or expecting a child (excluding 61 dyads), and (d) those not missing on key covariates (excluding 14 dyads). The final sample was 608 dyads (and, by default, 608 men and 608 women). The analytical sample exclusions are displayed in Fig. 1.

### Measures

#### Dependent variables

The main dependent variable is a couple-level measure of agreement on fertility desires. Each respondent was asked “Would you, yourself, want a(nother) child?” with response categories of definitely yes, probably yes, probably no, definitely no, and not sure. To create a couple-level measure to test Hypothesis 1, we first combined definitely and probably yes into one category and probably and definitely no into another category. Then we created a three-category joint measure: (1) both agree they probably/definitely want a(nother) child; (2) both agree they probably/definitely do not want a(nother) child; and (3) disagree on wanting a(nother) child or both are unsure. For this latter category, the vast majority (93%) are couples who disagree about wanting a(nother) child (rather than both are unsure). To test Hypothesis 2, which considers each partner’s individual desires as a function of their own stress and their partner’s relative stress, we do not collapse the variable, though we do recode it so that lower scores mean less desire to have a child and higher scores equate to more desire, with the midpoint representing uncertainty.

#### Independent variables

The key independent variables are three domains of pandemic-related stressors: health, economic, and relationship. For health and economic stress, all included items were prefaced with, “How stressed are you about the following?” Each item was reported on a scale from 1 (Not at all stressed) to 5 (Very stressed). Health stress was measured using the following items: (a) getting coronavirus, (b) my spouse or partner getting coronavirus, and (c) my parents, siblings, or other family members getting coronavirus. All three items were averaged for a total score, with higher scores representing higher levels of health stress (*a* = 0.89 for main respondents, *a* = 0.87 for partners). Economic stress was measured, again with question “How stressed are you about the following?” using the following items: (a) money and finances, (b) my job, and (c) getting food and supplies. All three items were averaged for a total score, with higher scores indicating higher levels of economic stress (*a* = 0.75 for main respondents, *a* = 0.70 for partners). Relationship stress was the third indicator of stress during the pandemic. Three items were used in this measure: (1) “Our relationship will be stronger than ever after the coronavirus pandemic is over”, (2) “The coronavirus pandemic is making me question my relationship”, and (3) “After the coronavirus pandemic is over, we will probably break up, separate, or divorce.” Responses to each item were recorded on a scale from 1 (strongly disagree) to 5 (strongly agree). The first item was reverse coded, and the three items were averaged for a total score, with higher scores representing higher relationship stress (*a* = 0.71 for both main respondents and partners). To test Hypothesis 1, for each type of stress, we created an average couple-level stress variable combining each respondents’ own stress. We could not enter each partner’s stress independently in the couple-level models as they were too highly correlated. To test Hypothesis 2, which considers each partner’s individual fertility desires, we use the partner’s own stress and a relative measure of whether their partner has lower, the same level, or higher stress, defined as one standard deviation above or below the average for that stressor.

We control for sociodemographic and economic characteristics. In the couple-level analyses testing Hypothesis 1, these include age of the youngest partner, whether the couple was married vs cohabiting,^2^ parity (0, 1 or 2 or more), joint education^3^ (both have a 4-year college degree; one has 4-year college degree; neither has a 4-year college degree), employment status (both work full-time; one works full-time and one works part-time; one works full-time and one does not engage in paid labor; neither working full-time)^4^; whether the respondent’s household had experienced one or more financial hardships in the past month (out of six items such as “we were unable to pay our gas, electric, other utility bill or rent/mortgage”, “we were unable to make minimum payment on credit cards”, “we received an eviction or foreclosure notice”), and race-ethnicity (both non-Hispanic White; both same race other than non-Hispanic White; interracial).

For the individual-level analyses run separately by gender to test Hypothesis 2, covariates include the respondent’s age, whether the union was a marriage vs. cohabitation, parity (0, 1, or 2 or more), a binary indicator of having at least a 4-year college degree, employment status (working full-time; working part-time; not working for pay); whether the respondent had experienced one or more financial hardships in the past month, and the respondent’s race-ethnicity (non-Hispanic White, non-Hispanic Black, Hispanic, Asian/Pacific Islander, some other race, or multiracial). Note that marital status, parity, and financial hardship experience in the last month are couple- or household-level and so do not differ from the measures described above. Weighted descriptive characteristics for the couple-level analyses are shown in Table 1 and for the individual-level analyses are shown in Table 2.

**Table 1:** Weighted percentages and mean/standard errors of the couple-level sample (*N* = 608)

Fertility desires	
Both want a(nother) child	22.1%
Both do not want a(nother) child	53.7%
Disagree/both unsure on wanting a(nother)child	24.2%
Economic stress	2.40 (0.05)
Health stress	2.76 (0.08)
Relationship stress	1.68 (0.04)
Age of youngest partner	35.9 years (0.67)
Marital status	
Cohabiting	17.9%
Married	82.1%
Parity	
None	30.0%
One	15.9%
Two or more	54.1%
Education	
Both bachelor’s degree	32.7%
One with bachelor’s degree	25.1%
Neither with bachelor’s degree	42.1%
Employment	
Both full-time	45.3%
One full-time, one part-time	14.8%
One full-time, one not working	32.1%
Neither full-time	7.8%
Financial hardship	21.1%
Couple race/ethnicity	
Both non-Hispanic White	53.6%
Both non-Hispanic, non-White same race	28.1%
Interracial	18.4%

**Table 2:** Weighted percentages and mean/standard errors of the individual-level sample

	Men (*N* = 608)	Women (*N* = 608)
Fertility desires		
Definitely want	16.2%	16.8%
Probably want	15.2%	13.0%
Unsure	7.6%	8.2%
Probably do not want	13.6%	12.3%
Definitely do not want	47.4%	49.7%
Economic stress	2.32 (0.06)	2.48 (0.06)
Health stress	2.56 (0.10)	2.96 (0.07)
Relationship stress	1.67 (0.04)	1.69 (0.05)
Partner’s relative stress		
Economic stress		
Same	65.8%	65.8%
Lower	16.4%	17.8%
Higher	17.8%	16.4%
Health stress		
Same	68.1%	68.1%
Lower	14.1%	17.8%
Higher	17.8%	14.1%
Relationship stress		
Same	83.0%	83.0%
Lower	9.9%	7.1%
Higher	7.1%	9.9%
Age	38.1 years (0.66)	36.5 years (0.70)
Employment		
Full-time	81.9%	55.7%
Part-time	6.2%	15.5%
Not employed	12.0%	28.9%
Educational attainment		
No bachelor’s degree	60.5%	48.9%
Bachelor’s degree	39.5%	51.1%
Race/ethnicity		
Non-Hispanic White	62.4%	60.8%
Non-Hispanic Black	7.6%	7.5%
Asian/Pacific Islander	8.0%	8.9%
Other race	0.7%	0.6%
Multiracial	3.0%	2.9%
Hispanic	18.2%	19.3%

Because the analyses are restricted to couples, shared characteristics of marital status, parity, and household financial hardships in the past month are not shown in this table since they are identical in distribution to those at the couple-level in Table 1

#### Analytical approach

We present the distribution of fertility desires and the mean level of each type of stress for couples and then for men and women separately. Next, to test Hypothesis 1, we estimate multivariable multinomial logistic regression models, showing relative risk ratios (RRRs) and presenting three contrasts: (1) both do not want vs both want, (2) disagree/both unsure vs. both want, (3) disagree/both unsure vs both do not want. RRRs above one indicate a positive association between a characteristic and one category of the outcome (i.e., both want) relative to another category outcome (i.e., both do not want), and RRRs below one indicate a negative association; an RRR of one indicates no association. To test Hypothesis 2, we run models separately for men and women, using ordered logit models to predict the respondent’s own fertility desires as a function of their own level of stress and their partner’s relative stress, controlling for the respondent’s own characteristics, showing odds ratios. Odds ratios from ordinal logistic regression can be interpreted similarly to odds ratios from logistic regression, where a value greater than one indicates a positive association with the outcome variable and a value less than one indicates a negative association. In this case, a value above one indicates greater fertility desire (and below one less desires) associated with a particular covariate. Because the stressors were highly correlated with each other, we present three models focusing on each stressor for both the couple-level and individual-level models (but no full model with all three stressors): Model 1 focuses on economic stress, Model 2 focuses on health stress, and Model 3 focuses on relationship stress. In each model, we control for sociodemographic covariates. All analyses are weighted (Marlar et al., 2022), though we do not use robust standard errors since heteroscedasticity was not present in the data, nor were the data clustered.

## Results

### Descriptive statistics

Table 1 shows the couple’s agreement on fertility desires and the couple’s average level of stress for the three stressors. Just over half (53.7%) of the couples agreed they did not want any (more) children. The remainder were fairly evenly split among those agreeing they did want a(nother) child, 22.1%, and disagreeing or both being unsure about having a(nother) child (24.2%). Thus, disagreement or uncertainty about fertility desires was not uncommon among these couples. Regarding mean couple-level stress across domains (recall that stress is measured on a scale of 1–5, with higher scores equating to more stress), health stress was highest, at 2.76, and relationship stress was lowest, at 1.68, with average couple-level economic stress at 2.40. Table 2 presents the same set of descriptive statistics separately for men and women as individuals. Because disagreement is not included, the individual-level perspective reflects greater certainty about wanting or not wanting a child relative to the couple-level perspective. We find few gender differences in reports of wanting, not wanting, and uncertainty about having a(nother) child.

### Multivariable analyses

Table 3 shows relative risk ratios (RRRs) from multinomial logistic regression predicting couple’s fertility desires and agreement. Model 1 focuses on economic stress and shows that higher levels of economic stress are statistically and significantly associated with increases in the risk of both members of the couple being unsure or disagreeing relative to either agreeing they want or agreeing that they do not want. Specifically, as economic stress increases by one unit, the relative risk of partners being unsure about or disagreeing about fertility desires doubles relative to agreeing on wanting (RRR = 2.03) and increases by about 75% relative to agreeing on not wanting (RRR = 1.77) a(nother) child. Model 2 shows that higher levels of health stress are statistically and significantly associated with being unsure or disagreeing relative to wanting a(nother) child (RRR = 1.81). Model 3 highlights relationship stress, and greater relationship stress is associated with increases in the risk of being unsure or disagreeing relative to wanting a(nother) child (RRR = 2.77). Overall, higher levels of stress across each domain are associated with higher likelihoods of couples’ reports of disagreement or uncertainty about fertility desires.

**Table 3:** Relative risk ratios from multivariable multinomial logistic regression of couple-level agreement on fertility desires

	Model 1			Model 2			Model 3		
	Not want vs. want	Disagree/unsure vs. want	Disagree/unsure vs. not want	Not want vs. want	Disagree/unsure vs. want	Disagree/unsure vs. not want	Not want vs. want	Disagree/unsure vs. want	Disagree/unsure vs. not want
Mean couple-level stress									
Economic stress	1.15	2.03**	1.77**						
Health stress				1.66	1.81**	1.09			
Relationship stress							2.08	2.77*	1.33
Youngest partner age	1.24***	1.12**	0.90***	1.23	1.12**	0.91***	1.23***	1.11**	0.90***
Married (ref: Cohabiting)	2.82*	3.86**	1.37	3.56	3.86**	1.08	3.23**	3.82**	1.18
Parity (ref: None)									
One	0.27**	0.28*	1.05	0.27	0.27*	1.03	0.27*	0.28*	1.03
Two or more	2.35	1.36	0.58	2.50	1.34	0.54	2.38	1.27	0.53
Bachelor’s degree (ref: Both partners)									
One partner	0.96	0.66	0.69	1.11	0.82	0.74	0.97	0.69	0.72
Neither partner	0.92	0.66	0.73	1.17	0.94	0.81	1.00	0.80	0.80
Employment (ref: Both full-time)									
One full-time/one part-time	0.62	0.38	0.61	0.73	0.48	0.65	0.63	0.38	0.61
One full-time/one unemployed	1.47	1.30	0.89	1.46	1.41	0.96	1.52	1.46	0.96
Neither full-time	0.60	0.61	1.01	0.62	0.71	1.15	0.71	0.83	1.17
Financial Hardship	1.16	2.55	2.21*	1.25	3.87**	3.10**	1.05	3.09*	2.95**
Couple Race/ethnicity (ref: Both non-Hispanic White)									
Both non-Hispanic non-White, same race	1.12	1.77	1.58	0.95	1.28	1.35	1.16	1.60	1.37
Interracial	0.87	1.47	1.69	0.76	1.27	1.66	0.87	1.44	1.66
*F*	5.91***			4.77***			4.51***		

^***^p < 0.001. **p < 0.01. *p < *0.05*

The sociodemographic covariates are generally consistent across models. As the age of the youngest partner increases, couples are more likely to agree they do not want a child relative to wanting a(nother) child or to be disagree/unsure relative to wanting a(nother) child but less likely to be unsure or disagree relative to not wanting a child. Compared to cohabiting couples, married couples are less likely to agree they both want a child relative to agreeing they both do not want a child or disagreeing/being unsure. Couples with one child are less likely to agree they do not want a child or disagree/be unsure relative to wanting another child compared to childless couples. Financial hardship increases the risk of disagreeing/being unsure relative to either both agreeing they want a(nother) child or both agreeing they do not want a(nother) child.

Tables 4 and 5 show the results of ordered logistic regression predicting partnered men’s and women’s fertility desires at the individual, respectively, as a function of their own stress and their partner’s relative stress. The fertility desires are coded such that higher scores equate to more desire. Models 1 and 2 in Table 5 do not support the hypotheses that men’s own economic stress nor health stress—or their partner’s relative economic or health stress—is associated with men’s own fertility desires. However, in Model 3, although men’s own relationship stress is unrelated to their fertility desires, having a partner whose relationship stress is higher than their own is statistically and significantly related to lower desire to have a(nother) child. Specifically, men who had a partner who reported higher relative stress had fertility desires one point lower on average than men whose partner reported a level of similar level of relationship stress. The covariates are largely consistent across models. As men’s age increases, fertility desires decrease, and married men have lower desire than cohabiting men. Men who have at least two children have lower fertility desires than childless men, and financial hardship increases fertility desires.

**Table 4:** Odds ratios from ordered logit regression models predicting partnered men’s fertility desires

	Model 1	Model 2	Model 3
Economic stress	0.08		
Health stress		−0.18	
Relationship stress			−0.14
Partner relative stress (ref: Same)			
Lower	−0.17	−0.24	−0.78
Higher	−0.33	−0.47	−1.18*
Age	−0.15***	−0.15***	−0.15***
Marital status (ref: Cohabiting)			
Married	−0.44	−0.49	−0.56*
Couple parity (ref: None)			
One	0.77	0.67	0.68
Two or more	−0.59	−0.65*	−0.67*
Education (ref: Less than bachelor’s)			
Bachelor’s degree	0.15	0.22	0.14
Employment (ref: Full-time)			
Part-time	−0.32	−0.14	−0.28
Not working	−0.15	−0.15	−0.07
Financial hardship (ref: No)			
Yes	0.73	0.82*	0.90*
Race/ethnicity (ref: Non-Hispanic white)			
Non-Hispanic Black	0.09	0.14	0.11
Asian/Pacific Islander	0.15	0.16	0.16
Other race	−0.43	−0.37	−0.48
Multiracial	−0.48	−0.58	−0.47
Hispanic	0.13	0.16	0.09
*F*	11.06***	10.77***	10.14***

^***^p < 0.001. **p < 0.01. *p < *0.05*

**Table 5:** Odds ratios from ordered logit regression models predicting partnered women’s fertility desires

	Model 1	Model 2	Model 3
Economic stress	0.002		
Health stress		−0.06	
Relationship stress			−0.10
Partner relative stress (ref: Same)			
Lower	−0.13	−1.03**	−0.57
Higher	−0.41	0.22	−0.32
Age	−0.13***	−0.14***	−0.14***
Marital status (ref: Cohabiting)			
Married	−0.60	−0.50*	−0.62
Couple parity (ref: None)			
One	1.07**	1.00**	1.01**
Two or more	−0.37	−0.42	−0.37
Education (ref: Less than bachelor’s)			
Bachelor’s degree	0.32	0.30	0.28
Employment (ref: Full-time)			
Part-time	0.77**	0.70*	0.71*
Not working	0.18	0.10	0.17
Financial hardship (ref: No)			
Yes	−0.03	−0.002	−0.02
Race/ethnicity (ref: NH/NL White)			
Non-Hispanic Black	0.22	0.25	0.21
Asian/Pacific Islander	0.20	0.30	0.23
Other race	1.05	1.34	0.87
Multiracial	0.25	0.35	0.28
Hispanic	0.03	0.08	0.004
*F*	6.98***	6.56***	6.16***

^***^p < 0.001. **p < 0.01. *p < *0.05*

For women (Table 5), we see no evidence that their own level of stress in any of the domains (economics, health, or relationship) is associated with their own fertility desires, as with men. However, for these women, their male partner’s relative health stress is important. Women who have a partner with less health stress than women themselves report have lower fertility desires compared to women with partners who have similar levels of health stress, contrary to Hypothesis 2. The covariates are largely consistent across models for women but differ slightly from what is observed for men. Age and marital status are similarly related to fertility desires among women as they are for men. Women at parity one have higher fertility desires than childless women. Employment status is significantly related to fertility desires for women, though financial hardship is not. For women, being employed part-time increases fertility desires significantly relative to working full-time.

## Discussion

Declining fertility rates in the years since the Great Recession—and exacerbated during the COVID-19 pandemic—have encouraged demographers to think beyond traditional approaches to explaining fertility behavior that rely on objective economic indicators. Perspectives that highlight the role of uncertainty and subjective well-being for fertility decision-making in high-income countries, such as the Narratives of the Future (NofF) framework (Vignoli et al., 2020a, 2020b), provide new avenues to explore, though most applications of this perspective have focused on the European context. The Traits–Desires–Intentions–Behavior (TDIB) model of fertility posits that couples’ and individuals’ fertility desires are directly influenced by their conscious and subconscious dispositions (Miller, 1994; Miller et al., 2004); we argue that evaluations of current and future well-being would align with the ‘traits’ portion of the TDIB. As such, examining fertility desires—rather than intentions, as has been the case in most prior work—provides additional insights into fertility decision-making.

In this paper, we contribute to the burgeoning literature in demography about uncertainty and stress in the U.S. context (Guzzo & Hayford, 2025; Manning et al., 2022) by considering couples’ agreement on fertility desires as a function of their joint stress in different domains. We used unique dyadic data that allow us to accurately measure couples’ agreement and relative stress by using direct reports from both members of a couple rather than use one person’s report of their partner’s characteristics, as has been done in other studies and is not always accurate (Stykes, 2018). Overall, about one-quarter of couples were uncertain or disagreed about their fertility desires. When considering the link between different types of stress and couple-level fertility desires, we found that American couples’ joint level of economic stress was strongly related to uncertainty and disagreement about whether they do, or do not, want a(nother) child, consistent with Hypothesis 1. That is, as economic stress increases, it seems couples find it difficult to decide one way or another—or come to an agreement—about their fertility desires. We find that fertility desires are related not only to economic factors but also stress in the health and relationship realms. Couples with higher levels of health stress or stress about their relationship also express more uncertainty or disagreement their fertility desires relative to both agreeing that they want a(nother) child. We also explored models separately for men and women within our couple sample to assess how relative stress operates; in these models, one’s own fertility desires were a function of their own level of stress and their partner’s relative stress. For both men and women, their own stress in different domains was unrelated to their fertility desires. However, we observed some important associations with partners’ relative stress. For men, although their own level of relationship stress was unrelated to their fertility desires, having a partner with higher relationship stress was associated with significantly lower fertility desires, as we hypothesized. For women, having a partner with lower health stress is associated with lower fertility desires, which is contrary to our expectation.

Our general conclusion from these results is that stress and uncertainty influence the formation of fertility desires but do so largely at the couple level. Recall that the majority—93%—of couples in the “disagreement or both unsure” category were those that disagreed; this suggests that partnered men and women react to stressors across domains in different ways that influence how they each formulate their desires to have a child or have additional children. This, in turn, leads to disagreement within a couple about future fertility desires, as expected. But the finding that for women, their partner’s lower relative health stress was associated with lower fertility desires was unexpected. Why might that be the case? Having a male partner who is less worried about health during the pandemic may suggest they were not taking public health precautions seriously, which may lead women to worry about whether their partner would take health care needs for their children as seriously as mothers would. It could also reflect the fact women may have been particularly aware of the restrictions in the U.S. related to whether the partners of pregnant people could accompany them to doctor’s visits or during delivery (Arora et al., 2020). For men, the only partner-level measure that mattered was whether their female partner had more relationship stress than they did. Women may be more attuned to their relationship’s quality and its likely longevity in ways that influence fertility goals.

Our work also provided support for the notion that stress and uncertainty is multidimensional. Prior work using the NofF framework has primarily focused on economic factors (Brauner-Otto & Geist, 2018; Gatta et al., 2022; Guetto et al., 2022; Lappegard et al., 2022; Vignoli et al., 2020b). We find that health stress and relationship stress were also significant for both couples’ and individuals’ fertility desires. This supports prior work that emphasizes a need to move beyond the economic realm (Comolli, 2023; Comolli et al., 2021; Manning et al., 2022) and makes a case for considering a wider range of factors that reflect concerns about the future.

### Limitations

We focused on couples’ desires as a function of their joint uncertainty and stress in different domains and considered gender-specific models, but we did not fully explore within couples which partner’s (men’s vs. women’s) uncertainty and stress were linked to fertility desires and agreement. The data are also cross-sectional, and so we are unable to establish causal relationships, nor can we identify which couples do (or do not) go on to have a child. It is also possible that other unmeasured factors, such as general personality traits, drive both uncertainty and fertility desires. Our analytical sample is also a subset of the larger NCHAT sample in that both the main respondent and their partner had to participate. It may be the case that such couples are more selective of those with stronger relationships (as suggested by the low levels of relationship stress indicated in Tables 1 and 2) or some other factor that increased joint participation and could also be linked to fertility goals. Arguably, the selectivity of the dyadic sample is offset by having accurate information about each partner’s fertility desires and stress rather than less accurate proxy measures. The survey collection and the key items of interest are specific to the COVID-19 pandemic; though we argue that the pandemic provided an opportunity to study uncertainty during a period of heightened social upheaval, the results may not be generalizable to either pre- or post-pandemic periods. NCHAT does not have measures of respondents’ experiences with accessing reproductive technologies, which could also be related to fertility decision-making (though more strongly with intentions than general desires). Finally, we focused only on different-gender couples. The process for having a child within a same-gender relationship is more complex, with a greater role for external influences—such as the closure of fertility clinics or finding a surrogate—that were affected by the COVID-19 pandemic. Moreover, our theories on decision-making within a couple have, to date, been built primarily around how power operates in different-gender couples; more work is needed to understand how same-gender couples approach childbearing.

It is also worth reiterating that our work focused on the U.S. The NoF framework has primarily been applied—and supported—in Europe, and our work supports the application of the NoF framework to other contexts. However, there are some unique aspects of the U.S. that may limit the generalizability of the findings here. In particular, the U.S. lacks universal health care, and its health system consistently underperforms its peer nations in meeting the needs of its population (Blumenthal et al., 2024). Health concerns could thus be especially salient for childbearing decision-making in the U.S. relative to other countries, though other work suggests that health is relevant for fertility goals even when there is a universal health care system (Lazzari & Beaujouan, 2025). Similarly, the lack of strong parental supports in the U.S. relative to other countries (Calarco, 2024; Collins, 2019) may make fertility decision-making among American couples particularly sensitive to economic and relationship stressors at the couple level. Future work should consider whether and how macro-level social supports could potentially mitigate the role of stress on fertility decision-making across contexts, potentially by engaging in cross-country comparative research.

## Conclusion

Building on the emphasis on uncertainty introduced by the NofF framework for understanding fertility decision-making in high-income contexts (Vignoli et al., 2020a, 2020b), our work demonstrates the importance of widening the lens beyond economic uncertainty as well as the importance of situating fertility decisions within couples. And while the data used were specific to the COVID-19 pandemic, the findings likely extend more broadly to how individuals and couples make sense of childbearing in an era where uncertainty across multiple dimensions seems to be a more permanent facet of contemporary life. Childbearing is inherently a future-oriented behavior. Other dimensions not considered here but likely important include environmental concerns and worries about safety from internal threats [such as discrimination against members of the LGBTQ community (Gustafson et al., 2025)] and external threats [such as war (Perelli-Harris et al., 2024)]. In the U.S., additional fears linked to the erosion of reproductive autonomy and high rates of maternal mortality provide other potential concerns for would-be parents. Thus, continued attention to how individuals and couples make sense of uncertainty and how they are linked to fertility decision-making is crucial. This attention should include deep dives into how specific populations—such as the LGBTQ + community and members of racially minoritized groups—experience uncertainty and how this influences their fertility goals.

## Data Availability

The dataset used in the analysis is available from the Data Sharing for Demographic Research repository. https://www.icpsr.umich.edu/web/DSDR/studies/38417

## Footnotes

1 In the U.S., only 1% of births occur to men 50 and older (Ha et al., 2024). Including those over 50 does not substantively change the results of our analyses (not shown).

2 Recall that all respondents were in a coresidential romantic relationship.

3 In the U.S., having a 4-year college degree (or not) strongly delineates a wide range of family behaviors (Lundberg, Pollak, & Stearns, 2016).

4 Recall that the survey was fielded during the pandemic, when many businesses laid off workers.
